# Dr. Scudamore on the Parisian Practice

**Published:** 1826-07

**Authors:** 


*Observations on M. Laennec's Method of Forming a Diagnosis of the Diseases of the Chest by means of the Stethoscope, and of Percussion; and upon some Points of the French Practice of Medicine.* By Charles Scudamore, M.D. F.R.S. &c. 8vo. pp. 123. London, April, 1826.

The different subjects touched upon in this small volume have come so often before us in this Journal, and have, in fact, been so much more fully discussed by ourselves than by Dr. Scudamore, that we shall be very brief indeed in our notice of the present production. We are glad that a metropolitan physician has, at last, publickly acknowledged his faith in auscultation, and that faith founded on actual observation of the practice as pursued by the gifted author of stethoscopy itself. Much prejudice, we are aware, obtains in this country, and especially in this metropolis, against the use of the stethoscope in the investigation of thoracic diseases, founded principally, we believe, on the sacrifice of time and application necessary for becoming a proficient in the study of mediate auscultation—a study which we acknowledge is not a very easy one, especially as regards the more minute phenomena disclosed through the medium of the stethoscope. We do not, therefore, much wonder that metropolitan physicians engaged in private practice, which almost all the hospital physicians are, should be averse to a new study like this, which requires time, not only to become acquainted with the principles, but to apply them at the bed-side to practice. The principal object is, to get through as great a number of patients as possible, and that in the shortest time. The common means of investigation are considered tedious enough, and quite adequate to all *useful* purposes—what more, therefore, is wanted?

Dr. Scudamore has, however, visited the Parisian hospitals, and studied stethoscopy under the first masters. We hope he will be successful in inspiring his colleagues of the metropolis to pursue the investigation. This is all we can do or say on the subject, as we have already dedicated more time and space to the dissemination of auscultic principles than any other journalists on this side of the channel. Our author has related some cases in his own private practice where the stethoscope has been usefully employed, but these cases we must refer to the reader's own perusal in the volume itself.

Neither can we dwell long on the other portion of our author's work, which touches on some points of French practice, as contrasted with English. We have given, and we daily give, so much of Parisian *therapeutics* unavoidably appended to *pathology* (the main object of our importation) that the English reader is thus brought, as it were, into the sick chamber, or the wards of the hospital, before he visits the dead-house or the dissecting-room.

Dr. Scudamore makes, we think, some judicious observations on the difference between French and English practice. We have, in various parts of this Journal, remarked that climate, diet, and modes of life, must modify the functions or even the structure of the human frame, so as to render it more or less susceptible of the operation of medicinal agents, and thus account for the different systems of therapeutics adopted by different nations.

"The general objection amongst the French to the use of calomel prevails almost as strongly as at former periods; and certainly amounts to a prejudice. I learnt, on good authority, at Paris, that this medicine is usually found to act in a peculiar manner on the constitution of the French patient; as it commonly produces irritation in the intestinal canal to a degree that causes extreme discomfort. I need not relate with what freedom and satisfaction the English make use of this active medicine.

"I apprehend that the difference of operation is to be ascribed chiefly to the respective nature of the diet in the two countries. The remark applies most particularly to the free use of light wines, amongst the French. The vin ordinaire must produce very different effects from the English malt liquor; or the use of plain or soda water, in conjunction with wines of more sound quality. Indeed the difference of regimen appears to me a sufficient explanation. I may here remark how very materially the action of mercurial medicine on the bowels is controlled by suitable preparation of the patient with mild mucilaginous drinks. If, on the contrary, the stomach and intestinal canal be charged with acescent liquor, vinous, or of any other kind, acescent food, and fresh fruits, irritation and disorder, more or less painful, may be expected as almost a certain consequence." 54.

There may be a good deal of truth in the above observations. The only thing positive which we know on this subject is, that during the late war, when such an immense number of French prisoners were confined in the prisons and pontons of this country, they were treated much in the same manner as English sailors are, when ill; nor did we observe this *national idiosyncrasy*, if we may use the expression, which seems to prevent the application of our common, and especially our mercurial medicines to the French constitution. But then, it must be observed, the patients were in our own climate, and living on English fare. This, of course, would make a considerable difference. At all events, in admitting that different nations require different modes of treatment, we are rather more liberal than the French. They condemn our therapeutics without reserve, and measure every thing by their own standard. This is not philosophical.

Dr. Scudamore animadverts on the universal practice in France of trusting to lavements rather than aperient medicines, properly observing, that when the digestive organs are out of order, especially the liver and duodenum, the effects of lavements are insufficient, "and he has witnessed examples in which serious complaint in the liver has made insidious progress, from the circumstance of the patient having placed reliance on this palliative relief, and neglected the employment of an effective course of medicine." Dr. Scudamore has no doubt of the frequent utility of applying leeches to the neighbourhood of the rectum, in various disorders of the abdominal viscera. Nature often points out the remedy by a determination to the hæmorrhoidal vessels. It is needless to remark, that local affections are best relieved by local bleedings.

How often have we reiterated the substance of the following sentiment.

> "In active inflammation of any of the important organs of the body, the *decided* conduct of the English practitioner in using the lancet as his chief remedy, promptly and boldly, and persisting till the dangerous force of the disease is conquered, demands, in my opinion, an undoubted preference over the more tardy, and I must add, the inefficient methods of the French. This leading difference in the method of treating dangerous inflammations, appears to me to constitute the most remarkable distinction in the practice of the English and the French physician."—63.

Dr. S. believes that there are fewer severe and dangerous diseases among the French than among the English, owing to the greater stability and dryness of their climate, lightness of their food, greater degree of exercise, and more lively disposition of the people. The *médecine expectante* is still very general in France, and to a certain extent, Dr. S. considers it judicious; nevertheless, he thinks it better to adopt the maxim, "*Venienti occurrite morbo*," than patiently look on the coming storm, without taking any means to disarm its force.

Broussais's doctrine is next adverted to by Dr. Scudamore, and nearly the same judgment is pronounced on it as that which we have done on several occasions. Our author finds great fault with M. Broussais for considering gout and rheumatism as the same disease—he might just as well identify small-pox and measles, because in both there is an eruption on the skin. Gout never assails the husbandman who unites temperance with his labours; but *he* is not exempted from rheumatism in any of its forms. Gout is the disorder of peculiar constitutions—of the adult age—of luxury in eating, intemperance in drink—and sedentary thoughtful habits. Rheumatism appears at every period of life, and in all kinds of constitutions. On the theory that gout is a *gastro-enterite*, with a development of irritation on the joints, the Broussaians prohibit purgative medicines, and apply leeches freely to the affected parts. Dr. S. admits, that in some circumstances of the gouty paroxysm, the mucous membrane is affected with inflammatory irritation, requiring suitable treatment; but, as a general principle of practice, he strongly advocates the use of alteratives and purgatives, and with only occasional exceptions, discountenances the local application of leeches.

As an exception to the timid practice of the French physicians, Dr. S. quotes M. Alibert, and M. Biett, at the Hospital St. Louis. These physicians give arsenical and other active preparations without apprehension. They prefer small doses of arsenical solution twice a day, for a continuance, to the use of large doses for a short time, in diseases of the skin. Tincture of cantharides, in the dose of twenty drops twice a day, is one of their favourite remedies, alternately with arsenic, in the treatment of the order squamæ.

"Subcarbonate of ammonia, dissolved in water, in the proportion of two drams to a pint and a half, and given in this quantity daily, is found useful in certain states of cutaneous irritation, apparently caused by a free employment of mercury for syphilis.

"The inveterate disease called *lupus* is very successfully treated by the application of an arsenical caustic. They allow the part to form a crust, before they apply a poultice or any emollient dressing.

"Neither expence nor trouble is spared in making the artificial medicated baths. The alkaline bath, prepared by dissolving the subcarbonate of soda in the proportion of two pounds to the necessary quantity of water, is extremely useful when the skin is affected with scales." 82

Dr. S. has witnessed Laennec's administration of tartar emetic, in the manner which we have more than once portrayed in this Journal. He verifies the statements published on this subject, tried their tartar emetic, and found it equal to our own. Finally, he has made some experiments in this country, (not carrying the doses so far as the Italians and French) and found it to answer with the English stomach. This circumstance will probably induce our hospital physicians in this country to give the practice a fairer trial than it has yet obtained.

Dr. Scudamore concludes his volume with some slight remarks on the various new medicines which have been lately introduced to practice through the instrumentality of the French chemists; but, for which we must refer the reader to the work itself. We shall close this short analysis by offering Dr. Scudamore our sincere thanks for his zeal in examining the Parisian hospitals, and for the candid and judicious observations which he has made on the practice there. We are gratified that they almost entirely coincide with those which we ourselves have long been in the habit of making, through the medium of this Journal.

